# AI-based preeclampsia detection and prediction with electrocardiogram data

**DOI:** 10.3389/fcvm.2024.1360238

**Published:** 2024-03-04

**Authors:** Liam Butler, Fatma Gunturkun, Lokesh Chinthala, Ibrahim Karabayir, Mohammad S. Tootooni, Berna Bakir-Batu, Turgay Celik, Oguz Akbilgic, Robert L. Davis

**Affiliations:** ^1^ Department of Internal Medicine, Section on Cardiovascular Medicine, Wake Forest University School of Medicine, Winston-Salem, NC, United States; ^2^ Quantitative Sciences Unit, Stanford School of Medicine, Stanford University, Stanford, CA, United States; ^3^ Center for Biomedical Informatics, UTHSC, Memphis, TN, United States; ^4^ Parkinson School of Health Sciences and Public Health, Loyola University Chicago, Chicago, IL, United States

**Keywords:** preeclampsia, electrocardiogram, ECG-AI, prediction, detection, gestational age, validation, AUC

## Abstract

**Introduction:**

More than 76,000 women die yearly from preeclampsia and hypertensive disorders of pregnancy. Early diagnosis and management of preeclampsia can improve outcomes for both mother and baby. In this study, we developed artificial intelligence models to detect and predict preeclampsia from electrocardiograms (ECGs) in point-of-care settings.

**Methods:**

Ten-second 12-lead ECG data was obtained from two large health care settings: University of Tennessee Health Science Center (UTHSC) and Atrium Health Wake Forest Baptist (AHWFB). UTHSC data was split into 80% training and 20% holdout data. The model used a modified ResNet convolutional neural network, taking one-dimensional raw ECG signals comprising 12 channels as an input, to predict risk of preeclampsia. Sub-analyses were performed to assess the predictive accuracy for preeclampsia prediction within 30, 60, or 90 days before diagnosis.

**Results:**

The UTHSC cohort included 904 ECGs from 759 females (78.8% African American) with a mean ± sd age of 27.3 ± 5.0 years. The AHWFB cohort included 817 ECGs from 141 females (45.4 African American) with a mean ± sd age of 27.4 ± 5.9 years. The cross-validated ECG-AI model yielded an AUC (95% CI) of 0.85 (0.77-0.93) on UTHSC holdout data, and an AUC (95% CI) of 0.81 (0.77-0.84) on AHWFB data. The sub-analysis of different time windows before preeclampsia prediction resulted in AUCs (95% CI) of 0.92 (0.84-1.00), 0.89 (0.81-0.98) and 0.90 (0.81-0.98) when tested on ECGs 30 days, 60 days and 90 days, respectively, before diagnosis. When assessed on early onset preeclampsia (preeclampsia diagnosed at <34 weeks of pregnancy), the model's AUC (95% CI) was 0.98 (0.89-1.00).

**Discussion:**

We conclude that preeclampsia can be identified with high accuracy via application of AI models to ECG data.

## 1 Introduction

Preeclampsia and hypertensive disorders of pregnancy are among the leading causes of maternal and infant morbidity and mortality in the world (1–5). More than 76,000 women die each year from preeclampsia and hypertensive disorders of pregnancy (1). Preeclampsia affects 3%–5% of pregnancies in the US. In addition about 16% of maternal deaths occurring in low- and middle-income countries are related to preeclampsia and eclampsia and are mostly attributed to limited medical care (6). Furthermore, late or delayed diagnosis or management of preeclampsia is associated with worse outcomes for the mother and infant (2, 7). Preeclampsia is characterized by elevated blood pressure during pregnancy, generally starting after 20 weeks of gestation (8). In these cases, elevated blood pressure has a direct effect on cardiovascular, renal and liver dysfunction (8, 9).

The relationship between hypertension and preeclampsia is complex and multi-directional: chronic hypertension is a risk factor for preeclampsia, and preeclampsia is associated with increased long-term future cardiovascular morbidity (including hypertension) and mortality in the mother (8, 9). Gene variants associated with cardiomyopathy are also associated with preeclampsia, and prolonged QT interval, altered *p*-wave duration, and LV strain are more common among females with preeclampsia compared to healthy pregnancies (10, 11). Infants with births complicated by preeclampsia are more likely to be premature, have intrauterine growth restriction and have an increased risk of death, resulting in up to 900,000 infant deaths per year (9, 12, 13). Identifying pregnant females at elevated risk for preeclampsia using low-cost tools may facilitate closer monitoring and timely interventions to reduce preeclampsia-related adverse events in both babies and mothers.

Low-cost screening tools and interventions are particularly important for assessment of maternal health during pregnancy globally, with even more benefits when made available in low and middle income countries (LMIC), with the overall aim of reducing maternal and fetal complications from preeclampsia and its cardiac-related comorbidities (1). Multiple clinical guidelines for diagnosis of preeclampsia exist (e.g., Preeclampsia community guidelines, PRECOG; National Institute for Clinical Excellence, NICE), but such guidelines are more tailored towards developed countries and often rely on clinical assessments (1). The World Health Organization (WHO) has highlighted the importance of mobile-based technologies and their advancement as important steps in detection and monitoring of preeclampsia to stratify care and deliver easily-accessible tools for decision making, especially in community areas with expensive, limited or inaccessible healthcare services (2).

Electrocardiograms (ECG) are simple, yet powerful data modalities that are relatively easy and inexpensive to obtain. Artificial intelligence (AI) applied to the raw digital data from 12 lead ECGs has shown ability to detect and predict risk for, cardiovascular conditions including atrial fibrillation, heart failure, and cardiomyopathy (10, 11, 14–16). Given the evidence for early and often severe cardiac involvement in females with preeclampsia, we hypothesized that the application of AI to digital ECG data could aid in the early identification of females with preeclampsia. In addition, such AI methods have the potential for global implementation due to the possibility of incorporation within smart portable devices. To our knowledge, this is the first study to use raw digital ECG data to detect preeclampsia.

## 2 Materials and methods

### 2.1 Study design and data sources

This retrospective matched cohort study was based on multi-center medical records. Patient inclusion criteria included limiting subjects to females 18 years or older at time of delivery, having had at least one ECG during pregnancy and diagnosed with preeclampsia (for cases). Females with ICD-9 and ICD-10 codes for preeclampsia (642.4×, 642.5×, 642.7× and O14.×) were selected as cases. Controls were matched on age at delivery ± 2 years, self-reported ancestry, and gestational age (± 2 weeks).

Digital ECG data and demographic information, including age, race, for both cases and controls between 2014 and 2020 were obtained from the electronic health records of the University of Tennessee Health Science Center/Medical Center in Memphis, Tennessee (UTHSC). This data was used for model building. Additionally, ECG and demographic data from Atrium Health Wake Forest Baptist (AHWFB) of cases and controls from 2001 to 2023 was obtained for external validation. Raw digital time-voltage 12-lead ECG data recorded at ten seconds were originally obtained during the routine provision of patient care during prenatal care visits or hospitalization of pregnant patients. Data for all patients and variables was complete, with no missing values.

This study was approved by the Institutional Review Boards (IRB) of the respective institutions and followed the Transparent Reporting of a Multivariable Prediction Model for Individual Prognosis or Diagnosis (TRIPOD) reporting guideline.

### 2.2 Deep learning model for preeclampsia

Data from UTHSC were split 80%–20% into training and hold-out datasets. The training data was used to build a preeclampsia detection model with five-fold cross-validation. This final model was then tested on the 20% hold-out data. A modified ResNet CNN, reported in Akbilgic et al. was used to predict the incidence of preeclampsia (17–19). The CNN algorithm uses one-dimensional (1D) ECG signal with 12 channels (each ECG lead being one channel) as an input. Dropout and regularization values were tuned to reduce risk of overfitting the ECG-AI model. For training, the batch size (the number of data points evaluated at a time to update the model hyperparameters) was set to 64 and training occurred over 100 epochs (the number of complete passes through the full training dataset). All model development and associated analyses were performed using the Python programming language.

In addition to internal validation on the 20% UTHSC hold-out data, the ECG-AI model was also externally validated on ECGs obtained from AHWFB. Evaluation of ECG-AI on both the hold-out and external validation data was done using the area under the receiver operating characteristics (ROC AUC), accuracy, sensitivity, specificity, and precision.

### 2.3 Subgroup analyses

Sub-analyses were performed to include: (i) women with preeclampsia who delivered at less than 37 weeks gestational age; and (ii) women with preeclampsia diagnosed at less than 34 weeks of pregnancy. DeLong's test was used to compare significant differences between AUCs of each subgroup.

### 2.4 Model validation on data from AHWFB

The ECG-AI was developed on data from UTHSC and validated using data obtained from the EHR at AHWFB. The same inclusion and exclusion criteria were applied. The five-fold cross-validated models from ECG-AI were deployed on the validation data and the outcomes were averaged as a final prediction. Evaluation metrics for the best operating models were assessed. The DeLong's test was used to test for any statistically significant difference in AUCs between the predictions from UTHSC data and the AHWFB external validation dataset.

## 3 Results

### 3.1 EKGs during pregnancy

The majority of ECGs were obtained prior to the diagnosis of preeclampsia, with only 12% of the ECGs taken at the time of diagnosis ±7days. The median time between ECG to diagnosis was 33 days. Because obtaining ECGs during prenatal care is not a consistent practice during routine obstetric care, we collected information on the reason for the ECG for the UTHSC cohort and compared the reasons among cases and controls (Supplementary Table S1). The main reasons for obtaining ECGs were chest pain and shortness of breath for both cases and controls—symptoms that occur frequently during pregnancy but are non-specific. Overall, controls had higher instances of syncope and dizziness compared to cases. It is important to note that the ECG model presented below was able to distinguish between females with and without preeclampsia, even though all women (cases and controls) had roughly equal occurrence and distribution of these non-specific symptoms.

### 3.2 Patient characteristics

**Figure 1:**
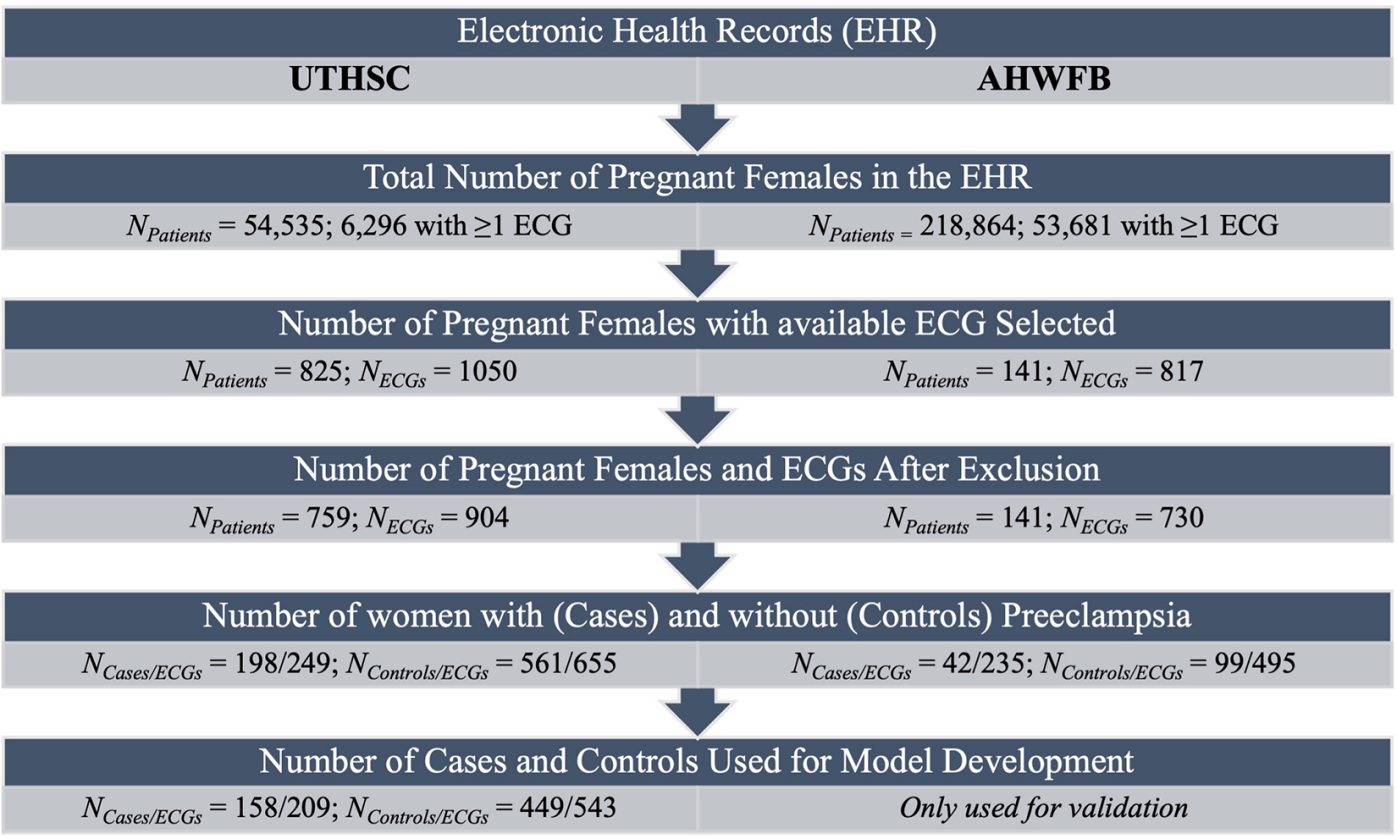
Flow chart summarizing number of patients and ECGs identified and included for model development.

The patient characteristics from UTHSC and AHWFB are shown in Table 1. The EHR at UTHSC included 54,534 pregnant women with 6,296 women having at least one ECG during pregnancy (a total of 9,895 ECGs). A total of 825 women from UTHSC were further identified, which then reduced to 759 after exclusion of women age <18 and/or ECGs of poor quality. From these, we identified 198 women with preeclampsia (cases) and 561 controls (See flowchart in Figure 1). The average age of cases and controls was 28.2 ± 5.8 and 27.0 ± 4.6 years, respectively. The make-up of the case group was 65.7% African-American and 29.2% white, while in the control group, 83.4% were African-American and 16.0% were white. For cases, the average gestational age at preeclampsia diagnosis and delivery was 34.8 ± 3.5 weeks and 36.0 ± 2.4 weeks, respectively. The mean gestational age at delivery was 37.4 ± 4.0 weeks for control women.

**Table 1:** Cohort characteristics of the UTHSC and AHWFB patient groups.

	UTHSC				AHWFB	
	Total *N*_Females _= 759 *N*_ECGs _= 904	Cases *N*_Females _= 198 *N*_ECGs _= 249	Controls *N*_Females _= 561 *N*_ECGs _= 655	Total *N*_Females _= 141 *N*_ECGs _= 817	Cases *N*_Females _= 42 *N*_ECGs _= 235	Controls *N*_Females _= 99 *N*_ECGs _= 495
Age mean ± sd	27.3 ± 5.0	28.2 ± 5.8	27.0 ± 4.6	27.4 ± 5.9	30.7 ± 6.3	27.4 ± 4.4
Race, *N* (%)						
African–American White Other/multiple	598 (78.8) 148 (19.5) 13 (1.7)	130 (65.7) 58 (29.2) 10 (5.1)	468 (83.4) 90 (16.0) 3 (0.5)	64 (45.4) 57 (40.4) 20 (14.2)	16 (38.1) 18 42.9) 8 (19.0)	48 (48.5) 39 (39.4) 12 (12.1)
GA/weeks at PE diagnosis, mean ± sd	–	34.8 ± 3.5	–	–	NA	–
GA/weeks at Delivery, mean ± sd	36.8 ± 4.1	36.0 ± 2.4	37.4 ± 4.0	NA	NA	NA
Co-morbidities and Complications Heart failure (yes) Hypertension (yes) Diabetes (yes) Gestational diabetes (yes) Multiple gestation (yes) Multiparity (yes) Stillbirth (yes) Cesarean delivery (yes)	5 (0.7) 87 (11.5) 58 (7.6) 43 (5.7) 29 (3.8) 5 (0.7) 18 (2.4) 223 (29.4)	1 (0.0) 42 (21.2) 21 (10.6) 23 (11.6) 15 (7.6) 0 (0.0) 6 (3.0) 88 (44.4)	4 (0.7) 45 (8.0) 37 (6.6) 20 (3.6) 14 (2.5) 5 (0.9) 12 (2.1) 135 (24.1)	NA	NA	NA

The EHR at AHWFB included 218,864 pregnant women with 53,681 pregnant women having at least one ECG taken during pregnancy.. Following the same selection protocol used to generate the cohort from UTHSC (including delivery information) resulted in a comparatively smaller sub-cohort from AHWFB. A total of 141 women (42 cases (with 235 ECGs) and 99 controls (with 495 ECGs total) from AHWFB were further selected to be included in the validation cohort The average age for cases and controls was 30.7 ± 6.3 years and 27.4 ± 4.4 years respectively. The case group was 38.1% African American and 42.9% white, while among controls, 48.5% were African American and 39.4% were white. Gestational information for AHWFB was unavailable.

### 3.3 Step 1: model evaluation, UTHSC

When applied to the 20% UTHSC holdout set, the cross-validation mean AUC (95% CI) was 0.85 (0.77–0.93) with an accuracy of 82%, precision of 63%, sensitivity of 78% and specificity of 84% (Table 2).

**Table 2:** Summary of evaluation metrics for the ECG-AI model tested on UTHSC 20% holdout data and on AHWFB validation data.

Evaluation metric	20% UTHSC holdout	AHWFB
AUC	0.85 (0.77–0.93)	0.81 (0.77–0.84)
Accuracy	82%	78%
Sensitivity	78%	66%
Specificity	84%	83%
Precision	63%	65%

### 3.4 Step 2: model evaluation, AHWFB external validation

The ECG-AI models developed on the UTHSC data were next evaluated on data from AHWFB. Validation of the ECG-AI models on AHWFB data resulted in an AUC (95% CI) of 0.81 (0.77–0.84) with accuracy of 78%, precision of 65%, sensitivity of 83% and specificity of 66%. Table 2 summarizes the evaluation metrics from the UTHSC holdout and AHWFB validation data sets.

### 3.5 Subgroup analyses

Additional analyses on the UTHSC holdout data (Table 3) were performed for model performance when limited to ECGs obtained within 30, 60, or 90 days before diagnosis, as well as when limited to ECGs obtained at least 30 days before diagnosis (i.e., excluding any ECG obtained within the month before diagnosis). Model evaluation resulted in AUCs (95% CI) of 0.92 (0.84–1.00) at 30 days, 0.89 (0.81–0.98) at 60 days and 0.90 (0.81–0.98) at 90 days before diagnosis. The AUC (95% CI) was 0.79 (0.66–0.92) when the model was tested on ECGs at least 30 days prior to diagnosis.

**Table 3:** Results on the 20% UTHSC holdout data for different time periods from diagnosis of preeclampsia or from delivery.

Sub-analysis on UTHSC 20% holdout	AUC	DeLong test (*p*-value)
ECGs taken ±30 days from first PE diagnosis	0.92 (0.84–1.00)	–
ECGs taken ±60 days from first PE diagnosis	0.89 (0.81–0.98)	–
ECGs taken ±90 days from first PE diagnosis	0.90 (0.81–0.98)	–
ECGs taken 30 + days before PE diagnosis^a^	0.79 (0.66–0.92)	–
PE developed when delivery was <37 weeks GA	0.76 (0.58–0.95)	0.219
PE developed when delivery was 37 + weeks GA	0.88 (0.77–0.99)	
PE diagnosed at <34 weeks of pregnancy	0.98 (0.89–1.00)	0.298
PE diagnosed at 34 + weeks of pregnancy	0.89 (0.75–1.00)	

PE, preeclampsia; GA, gestational age.

^a^ ECGs taken in the first month before diagnosis were excluded.

When we stratified by gestational age at delivery, the model had an AUC (95% CI) of 0.76 (0.58–0.95) among women with preeclampsia delivering at less than 37 weeks, and an AUC of 0.88 (0.77–0.99) for women delivering at 37 weeks or greater (no significant difference in AUCs; DeLong test *p*-value 0.219). When tested on women with preeclampsia diagnosed at less than 34 weeks gestational age the model AUC was 0.98 (0.89–1.00), and when evaluated on women with preeclampsia diagnosed at 34 weeks or greater gestational age, the AUC was 0.89 (0.75–1.00) (DeLong test *p*-value 0.298).

We also performed subgroup analysis of model performance among African American and white women. The model AUC (95% CI) was 0.83 (0.80–0.87) and 0.82 (0.76–0.88) for African American women from UTHSC and AHWFB, respectively (DeLong *p*-value = 0.724). Similarly, among white women, model AUCs (95% CI) were 0.85 (0.79–0.92) and 0.79 (0.74–0.84) on UTHSC and AHWFB, respectively (DeLong *p*-value = 0.161). There were no significant differences in model performance between African American and white women either at UTHSC or AHWFB (DeLong *p*-values of 0.433 and 0.437, respectively). Subgroup analyses were performed on women with a previous diagnosis of hypertension. When tested on women with a prior diagnosis of hypertension, the model AUC was 0.68 (0.45–0.91). When women with a previous diagnosis of hypertension were excluded from the dataset, the model AUC was 0.90 (0.82–0.98).

## 4 Discussion

In this study we show that ECG data can help identify pregnant women at high risk for preeclampsia. Our CNN model used 250 Hz raw 12-lead ECGs to classify and predict risk of preeclampsia with a cross-validated AUC of 0.85 on UTHSC data followed by an AUC 0.81 from our external validation (AHWFB) site, results comparable to other AI-based methods that utilize more detailed information (including laboratory testing) within machine learning algorithms (9, 20). The ECG-AI model showed good performance in predicting preeclampsia (AUC 0.89–0.92) between 30 and 90 days prior to the diagnosis. These findings open the possibility for ECG-AI use in smartwatches or similar mobile devices, which routinely capture single-lead ECG data, for remote monitoring of women at high risk during pregnancy.

Substantial research has been undertaken to identify females at high risk for preeclampsia, preferably with low-cost tools that can be used widely. Groups that have evaluated the utility of clinical and laboratory biomarkers to assess risk of preeclampsia (21–24) have reported moderate-high results (AUC between 0.80 and 0.90) when using such data within machine learning or neural network algorithms as shown by Jhee et al., Marić et al., Neocleous et al. and Li et al. (8, 9, 20, 25) At the same time, there has been considerable interest and research into the role of PlGF and sFlt-1 in preeclampsia testing (26). Approaches based on models using PlGF alone or the ratio of sFlt-1/PlGF have shown good performance and have been implemented for short term preeclampsia risk prediction and for assisting preeclampsia diagnosis in the second and third trimesters. First trimester screening performance is improved significantly when maternal history is combined with biophysical and biochemical results measured through pregnancy, including uterine artery pulsatility index (PI), mean arterial pressure (MAP), PlGF, and pregnancy-associated plasma protein-A (PAPP-A) (27). While promising, these models fundamentally differ from our approach, as they incorporate data that depend on blood analyses for biomarkers typically at a few points in time and that are not largely in clinical use (25).

As part of our work, we assessed model performance for ECGs obtained 30, 60 and 90 days before preeclampsia diagnosis. As would be expected, the highest AUCs were obtained closer to the diagnosis (AUC of 0.92 within 30 days of diagnosis) and remained high up to 90 days before diagnosis (AUC of 0.90). There was a decrease in AUC (0.79) when ECGs in the month prior to diagnosis were excluded. While this could mean that markers of preeclampsia manifest more within the 30 days closer to the diagnosis (12, 20), our model still operates well up to 90 days prior to diagnosis, at earlier stages of pregnancy, with increased potential for patient monitoring and clinical follow-ups (12, 16, 28).

We also assessed the model performance for subgroups of particular interest. For example, pregnant females of African-American ancestry are at higher risk of preeclampsia (29, 30). Our model performed equally among African-Americans and whites, with AUCs of 0.82 and 0.83, respectively. In addition, our model performed well [AUC of 0.98 (0.89–1.00)] in the detection of early-onset PE (diagnosed before 34 weeks).

We explored differences in women correctly identified as cases (true positives) vs. those misidentified as controls (false negatives). In the UTHSC holdout dataset, nine of the women with preeclampsia were misidentified as controls and of these, the majority had an ECG due to reported chest pain. Women correctly identified as having preeclampsia had an ECG due to history of hypertension or symptoms such as shortness of breath. While chest pain is associated with preeclampsia, as others have noted in some cases it might have inadvertently misled the clinicians and ultimately been associated with missed detection of preeclampsia (31, 32). Additionally, those correctly identified as cases (true positives) had more severe preeclampsia (with complications of childbirth) while women with missed preeclampsia diagnosis (false negatives) were more likely to have had preeclampsia diagnosed in the third trimester with no complications.

Five out of nine women with a previous history of hypertension who were misclassified as controls, (i.e., false negative) also underwent a cesarean delivery. This could mean that these women might have either undiagnosed or did not develop preeclampsia due to cesarean delivery, which might have been performed to reduce the risk of developing preeclampsia since these women were already at high risk (33, 34).

Early risk prediction for preeclampsia can allow for lifestyle intervention strategies, such as diet or physical activity (35, 36). Other interventions for high risk women include the use of pharmacological therapy, including the prescription of low dose Aspirin (37, 38). Our models perform better when the ECG was obtained within 90 days of diagnosis, which still allows time for either lifestyle or pharmaceutical interventions to reduce the risk for adverse maternal or infant outcomes. However, further model development, especially with larger and multi-institute datasets, is needed to identify the optimal performance windows.

Prior research with ECGs included signal processing methods to extract ECG features for use within machine learning methods. Such methods include wavelet transformation and probabilistic symbolic pattern recognition and can also be used not only for signal processing and feature extraction but also to reduce noise or artifacts (39–45). Our team had previously developed a similar method to diagnose preeclampsia, which used and compared signal processing methods for ECGs. Our preliminary results showed a slight, yet non-significant, increase in accuracy when using signal processing methods, in combination with extracted ECG features within machine learning algorithms (e.g., extreme gradient boosting). However, this method required additional processing steps, which reduces usability within clinical workflows. Therefore, the trade-off for simplicity using raw ECGs, with virtually no additional processing, within CNNs was preferred to increase simplicity. The current CNN structure can increase usability, application and implementation within the clinical workflow without relying on additional processing methods that can be time consuming and/or computationally expensive to implement.

Our results have implications for the use of ECG-based AI models, which are simple and cheap-to-execute and can also be embedded within point-of-care technologies (46). Portable 12-lead ECG monitors can be used this purpose, the data from which can be remotely collected and transferred to and from smart devices (47–49). There is also potential in developing single-lead ECG-based models for remote monitoring using smart wearables for pregnancies, especially among high risk women. We have previously shown that single-lead models perform well for the prediction and detection of heart failure (19, 46) and fatal coronary heart disease (50) using solely Lead I of a 12-Lead ECG, which is mimicked by smart watches and other smart devices with ECG monitoring capabilities. A similar approach can be taken for preeclampsia risk assessment. Since the models in this research use 12-Lead ECGs, our goal is to eventually develop and validate a single-lead ECG model (using Lead I of the ECG) that can be easily used in resource poor settings, and therefore focus on developing a model that requires no user input. However, future improvements to the model will consider including demographic characteristics such as age, height and weight.

This study has some limitations. The ECG-AI models were developed and tested on a dataset with mostly African-American and White patients and requires further development and testing on a more racially diverse cohort. While the models developed in this research were externally validated with similar results to the holdout-data, the models may not have leveraged the full potential of ECG in preeclampsia detection and risk prediction due to the limited sample size and relative lack of diversity beyond African-American and white women. A larger, racially diverse cohort could increase our models' predictive power and improve its generalizability to the general population and represents a future direction for research. In addition, while most clinical workflows are advancing to include AI to help with decision making, there are still issues that are to be considered, with most are related to standardizing its utility within clinical systems as well as their explainability (51).

## 5 Conclusion

In conclusion, our research shows that ECG-based models can detect women at high risk for preeclampsia with high accuracy. The simplicity of these models allows for integration within clinical workflows to help guide clinicians and/or patients to obtain further evaluation. By validating our models on an independent dataset from a different healthcare organization, this research shows the applicability of the ECG-based models across multiple healthcare institutions and its potential for remote monitoring.

## Data Availability

The data analyzed in this study is subject to the following licenses/restrictions: Data from University of Tennessee Health Science Center EHR and data from Atrium Health Wake Forest Baptist was used. Due to the nature of EHR, regulations apply. Requests to access these datasets should be directed to UTHSC: lchintha@uthsc.edu and AHWFB: CTSIdata@wakehealth.edu.
