# Supplementary material for: AI-based preeclampsia detection and prediction with electrocardiogram data
Source: Front Cardiovasc Med. 2024 Mar 4;11:1360238. doi: 10.3389/fcvm.2024.1360238 (PMC10945012; doi:10.3389/fcvm.2024.1360238)
Supplement: Supplementary file 1 [file Table1.docx]

**Table S1** Underlying indication for ECGs taken for controls and cases admitting to UTHSC

| **Reason for ECG** | **Total (N = 759)** | **Cases (N = 198)** | **Controls (N = 561)** |
| --- | --- | --- | --- |
| Chest Pain, N (%) | 236 (31.1) | 64 (32.3) | 172 (30.7) |
| Shortness of Breath, N (%) | 74 (9.7) | 18 (9.1) | 56 (10.0) |
| Syncope, N (%) | 55 (7.2) | 9 (4.5) | 46 (8.2) |
| Dizziness, N (%) | 54 (7.1) | 4 (2.0) | 50 (8.9) |
| Hypertension, N (%) | 35 (4.6) | 17 (8.6) | 18 (3.2) |
| Tachycardia, N (%) | 11 (1.4) | 5 (2.5) | 6 (1.1) |
| Palpitations, N (%) | 11 (1.4) | 2 (1.0) | 9 (1.6) |
| Miscellaneous*, (N) %) | 94 (12.4) | 36 (18.2) | 58 (10.3) |
| Not recorded, N (%) | 189 (25.0) | 43 (21.7) | 146 (26.0) |
| *Miscellaneous includes: abdominal pain, rapid heart rate, weakness, arrhythmia/dysrhythmia, numbness, arm pain, stroke, back pain pre-operative assessment, bradycardia, lightheadedness, vomiting, abdominal pain, skipped or accelerated heartbeat, bleeding, angina, edema, diabetes, nosebleeds, impaired renal function, palpitations, dyspnea, anxiety and/or excessive coughing | | | |
